# A Multi-Target and Multi-Channel Mechanism of Action for Jiawei Yinhuo Tang in the Treatment of Social Communication Disorders in Autism: Network Pharmacology and Molecular Docking Studies

**DOI:** 10.1155/2022/4093138

**Published:** 2022-02-08

**Authors:** Zhang Linlin, Lai Ciai, Su Yanhong, Gan Huizhong, Li Yongchun, Yang Zhen, Xu Shan, Gong Fengying, Lv Ying, Li Jingjun, Fan Qin

**Affiliations:** ^1^The Second School of Clinic Medicine, Guangzhou University of Traditional Chinese Medicine, Guangzhou 510410, China; ^2^Southern Medical University, Nanfang Hospital, Department of Ancient Traditional Chinese Medicine, Guangzhou 510610, China; ^3^School of Traditional Chinese Medicine, Southern Medical University, Guangzhou 510515, China

## Abstract

**Background:**

Autism spectrum disorder (ASD) is a highly heterogeneous neurodevelopmental disorder with complex pathogenesis. Currently, the pathogenesis of ASD is not fully understood. Moreover, current treatments do not effectively alleviate the primary symptoms of ASD social disorder (SCDA). Jiawei Yinhuo Tang (JWYHT) is an improved version of the classic prescription Yinhuo Tang. Although this medication has been shown to improve social behavior in ASD patients, the mechanism by which it works remains unknown.

**Methods:**

In this study, network pharmacology bioinformatics analysis was used to identify the key targets, biological functions, and signal pathways of JWYHT in SCDA. Then, molecular docking and molecular dynamic simulation were used to validate the activity and stability of the active ingredient and the target protein during the binding process.

**Results:**

The analysis identified 157 key targets and 9 core targets of JWYHT (including proto-oncogene (FOS), caspase 3 (CASP3), mitogen-activated protein kinase-3 (MAPK3), interleukin-6 (IL6), mitogen-activated protein kinase-1 (MAPK1), tumor necrosis factor (TNF), mitogen-activated protein kinase-8 (MAPK8), AKT serine/threonine kinase 1 (AKT1), and 5-hydroxytryptamine receptor 1B (5HT1B)) in SCDA. In addition, the Kyoto Encyclopedia of Gene and Genome results, as well as the staggering network analyses, revealed 20 biological processes and 20 signal pathways targeted by JWYHT in SCDA. Finally, molecular docking analysis was used to determine the binding activity of the main active components of JWYHT to the key targets. The binding activity and stability of methyl arachidonate and MAPK8 were demonstrated using molecular dynamics simulation.

**Conclusion:**

This study demonstrates that JWYHT regulates neuronal development, synaptic transmission, intestinal and cerebral inflammatory response, and other processes in SCDA.

## 1. Introduction

ASD is a clinical condition characterized by a set of neurodevelopmental symptoms. ASD is defined by three main characteristics: persistent impairment in social communication and interaction, repetitive behavior, and restricted interests [1]. The most recent study estimates the global prevalence of ASD to be 0.62% [2]. ASD is a chronic, lifelong disease that cannot be reversed [3]. The etiology of ASD has yet to be determined. Pharmacological intervention, comprehensive education, rehabilitation training, sensory integration, and nutrition therapy, on the other hand, are by far the most frequently used therapeutic approaches. Regrettably, these therapies have no clinically established therapeutic impact on the fundamental characteristics of ASD. Herbal medicine and acupuncture, a type of complementary and alternative medicine (CAM), have gained popularity as a therapeutic option for ASD children due to fewer potential adverse drug effects [4]. The number of RCT studies on herbal medicine is comparable to those on conventional therapy, demonstrating that herbal medicine is widely prescribed and recognized worldwide [5].

JWYHT is a traditional Chinese formula made up of Morindae officinalis Radix, *Schisandrae chinensis* Fructus, *Poria cocos*, Ophiopogonis Radix, Rehmanniae Radix Praeparata, Asparagi Radix, and Mume Fructus. JWYHT was first mentioned in the famous ancient medicine treatise, “Bian-Zheng-Qi-Wen,” of the Qing dynasty. Morindae Officinalis Radix has the function of strengthening sinew and bone, tonifying kidney yang, and dispelling wind-dampness in this Chinese formula. Rehmanniae Radix Praeparata is used to tonify blood, essence, and marrow as well as to enrich yin. *S. chinensis* Fructus is responsible for enriching qi, generating fluid, securing and tonifying the kidney, and nourishing the heart to tranquilize. *P. cocos* is primarily used to induce diuresis to drain dampness, fortify the spleen, and nourish the heart to tranquilize. Ophiopogonis Radix is frequently used to enrich yin, generate fluid, moisten the lung, and clear heartburn. Asparagi Radix is used to enrich yin, moisten dryness, clear lung fire, and generate fluid. Asparagi Radix is used to enrich yin, moisten dryness, clear lung fire, and generate fluid. Mume Fructus is used to astringe the lung, and intestine, as well as to calm Ascaris and stimulate fluid production. The whole formula feeds yin while tonifying yang. Pharmacologic studies have shown the positive therapeutic effects of certain components of JWYHT on ASD patients. *S. chinensis* Fructus and its active ingredients, for example, have demonstrated anti-oxidation, apoptosis suppression, anti-inflammation, and neurotransmitter properties. These modifications made modulating brain-derived neurotrophic factor (BDNF) related pathways easier [6]. Additionally, *Rehmannia glutinosa* can alleviate scopolamine-induced neuronal damage and memory dysfunction in rats, demonstrating a significant neuroprotective effect [7]. Currently, no certified research has examined the main mechanisms and functions of the complete JWYHT formula, as well as the underlying therapeutic mechanism when applied to ASD, leaving room for further investigation and discussion.

Traditional Chinese medicine (TCM) is a valuable medication that has been passed down through Chinese culture. It has evolved into a significant player in mainstream medicine. TCM evaluates a patient's symptoms and restores the balance of life and bodily functions by prescribing therapeutic herbs based on “King, Vassal, Assistant and Delivery servant” compatibility [8]. In recent years, TCM has played a significant role in clinical practice because of its low toxicity and potential therapeutic benefits [9–11]. Syndrome differentiation and therapy are the fundamental disease diagnosis and treatment system of TCM, and TCM is primarily demonstrated through traditional Chinese medicine preparation [12]. Various traditional medicine (TM) prescriptions have clinically proven effectiveness and acceptable therapeutic qualities [13]. This opens up a lot of new avenues for novel drug development in TMs.

In recent years, there has been an increase in the number of studies focusing on the advancement of network pharmacology in Chinese medicine. Based on these biological databases and clinical trial results, researchers may use systems biology to investigate the network connection between “herbs-compounds-proteins/genes-disease” and scientifically explain the mechanism of action of herbal medicines on specific diseases. The significance of network pharmacology is that it allows for the development of new ideas and approaches to drug research. As a result, biological systems, drugs, and diseases may be linked in a reliable network, benefiting the modernization and internationalization of TCM [12].

Herein, we used a network pharmacology-based approach, followed by molecular docking analysis, to identify the active ingredients, potential targets, and pharmacological mechanism of JWYHT in SCDA treatment. First, we searched for information on bioactive compounds in JWYHT. Candidate target genes for such compounds and SCDA were identified and collected from relevant databases. The drug ingredients-target network was constructed using the target sets of JWYHT and SCDA. The overlapped genes were examined using comparative analysis. Following that, protein-protein interaction (PPI) and module networks were built using overlapping genes and hub nodes, followed by GO and KEGG pathway enrichment analysis. Therefore, this study aimed to explore and validate the molecular mechanisms and pathways of the major bioactive components of JWYHT in SCDA treatment. This would provide a necessary and fundamental basis for further research into the pharmacological mechanism of JWYHT.

## 2. Materials and Methods

### 2.1. Screening for Active JWYHT Targets

The Traditional Chinese Medicine Systems Pharmacology (TCMSP, http://lsp.nwu.edu.cn/) and Traditional Chinese Medicine Integrated Database (TCMID, http://119.3.41.228:8000/tcmid/) were used to find well-reported pharmacological targets of JWYHT such as Morinda officinalis, Fructus schisandra, *P. cocos*, Praeparata Radix Rehmanniae, asparagus, black plum, and *Ophiopogon japonicus*. According to the pharmacokinetic absorption, distribution, metabolism, and excretion parameters, the criteria for screening out the active ingredients from the TCMSP were oral bioavailability (OB) ≥30% and drug-likeness (DL) quality ≥0.18 [14]. The active ingredient targets were then obtained from the PubChem database. The targets were identified using the SwissTargetPrediction database and the UniProt database (after removing duplicates). Simultaneous searches for SCDA pathological targets were conducted in GeneCards (https://www.genecards.org/) and Online Mendelian Inheritance in Man (OMIM, https://omim.org/). Using JWYHT drug targets and SCDA pathological targets as two independent sets, the intersection genes were obtained using the jvenn online software mapping tool platform. A Venn diagram was used to evaluate all of the collected targets and identified the ones that overlapped as potential therapeutic targets.

### 2.2. Constructing Related Targets Network of Treating SCDA by JWYHT

The Search Tool for the Retrieval of Interacting Genes/Proteins (STRING, https://string-db.org/) database was used to investigate the relationship between the targets. The species was set to “*Homo sapiens*,” and the lowest interaction threshold was set to 0.4. All other parameters were set to default. The protein-protein interactions of the JWTHT- and SCDA-related targets were exported, following which the tsv data was extracted. Cytoscape3.8.2 software was used to develop a medicine–component–disease-target network model based on the common targets for a better visual comprehension of their interrelationships. The major active targets of JWYHT were analyzed by degree value, revealing the number of components and action targets. The higher the degree value, the more powerful the biological function and effect of the ingredient.

### 2.3. Key Subnetwork Analysis and Structuring

Importing tsv of PPI data into Cytoscape 3.8.2 for key subnetwork analysis was performed in two steps. The first step was to use the CytoNCA network analyzer to compute the scores of betweenness (BC), closeness (CC), degree (DC), eigenvector (EC), local average connectivity-based method (LAC), and network (NC). Genes with scores higher than the median of the aforementioned items were successively selected to construct the final key subnetwork. The top 10 genes in PPIs and important subnodes that were independent by phase one were identified using cytoHubba chrome. MCODE can also be used to filter subsets of the entire PPI network.

### 2.4. Analysis of Functional Process and Molecular Pathways

The *R* software packages “DOSE,”“clusterProfiler,”“EnrichPlot,” and “PathView” were installed via the “BiocManager” and used to identify biological processes (BP), molecular functions, and KEGG pathways with *P*-values <0.05 and *Q*-value <0.05. This data was used to generate bubble graphs, histograms, and related route graphs.

### 2.5. Kyoto Encyclopedia of Genes and Genomes (KEGG) Pathway Analysis and Network of Visualization of Targets

KEGG-GENE visualization was performed in Cytoscape 3.8.2 software to identify potential targets and JWYHT-associated enrichment pathways for SCDA treatment. The connection between them could be easily demonstrated.

### 2.6. Core Target Screening and Molecular Docking Analysis

The most significant targets and active components with a degree >25.2 (twice the mean) were selected from the aforementioned essential subnetworks using a combination of literature mining and network analysis. The UniProt database (https://www.uniprot.org/) was used to obtain the PDB files of the three-dimensional structure of the receptor protein of the key genes. The files containing the two-dimensional structure of the main active ingredients were downloaded from the PubChem database (https://pubchem.ncbi.nlm.nih.gov/). The ChemBio3D software was then used to calculate and export the three-dimensional structure of the ingredients as mol files, which were subsequently imported into the SYBYL-X 2.0 software for dehydration and hydrogenation. Minimize module was used for molecular energy optimization. The results were saved as mol2 files. The PDB files were imported into SYBYL-X2.0 software, where we exposed the active site of the ligand, removed the crystal water, hydrogenated, removed the side chain residues, and optimized the energy using the AMBER7 FF99 force field. The remaining parameters were set to default. Next, the docking files containing active pockets were generated. Finally, we used the Surflex-Dock Screen docking mode of SYBYL-X2.0 software to batch dock nine JWYHT compounds and nine SCDA key target proteins and obtained the corresponding total score value. The matching method of this docking tool is primarily based on structural and shape similarity, and the benefits include high accuracy, a high positive rate, and efficient matching. Total score is a scoring function that uses negative logarithms to simulate the binding capacity. It converts the binding free energy formula (∆*G*0 = −2.303 RT × Total Score, where *R* denotes the ideal gas constant of the molecule and *T* denotes the thermodynamic temperature of the ideal gas). It is widely assumed that the lower the free binding energy of the receptor and ligand, the greater the stability, and the higher the total score value. When total score is >5, we considered it to have well binding activity, and when total score is >7, we considered it to have superactivity due to the combination of the receptor protein and molecular ligand [14].

### 2.7. Molecular Dynamics (MD) Simulation

The methyl arachidonate and MAPK8 with the highest total score were selected for molecular dynamics simulation. The semiflexible docking of methyl arachidonate and MAPK8 was performed again using the Glide software and LigandDocking to obtain the small molecule-protein complex system that was used as the initial structure for all-atom molecular dynamics simulation. First, the charge of small molecules was calculated using the antechamber module and Gaussian 09 software [15, 16]. After that, small molecules and proteins were described using GAFF2 and ff14SB force fields [17, 18], respectively. Following a series of operations such as the hydrogenation with the LEAP module, the addition of the octahedral TIP3P solvent box [19], and the Na^+^/Cl^–^ balance charge, molecular dynamics simulation was performed using the AMBER 18 software [20]. The system was optimized for energy before the simulation and then slowly heated to 298.15 K. The NVT system was then simulated at 500 ps, followed by an equilibrium simulation at 500 ps that was performed under NPT conditions. Finally, a 50 ns NPT system simulation with periodic boundary conditions was performed on the system. The change of the root-mean-square deviation of the complex system over time reflects the movement of this complex. The binding free energy is calculated using the MM/GBSA method [21–24] during the simulation process, and the change in the pose (conformation) of the binding mode of methyl arachidonate and MAPK8 before and after MD was visualized using PyMOL software.

## 3. Results

### 3.1. Active Ingredients and Potential Targets

**Figure 1:**
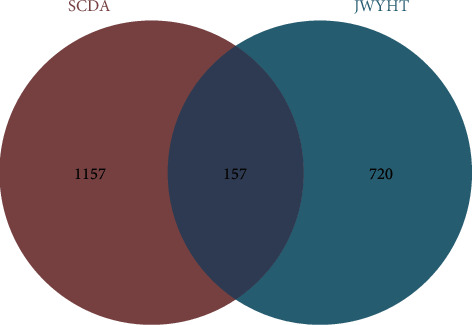
Drug-target disease-related genes were identified via the intersection of drug-target genes and SCDA-related genes.

The potential active components of Morinda Officinalis, Fructus schisandra, *P. cocos*, Praeparata Radix Rehmanniae, Asparagus, black plum, and *O. japonicus* were obtained by TCMSP and TCMID. A total of 877 drug target genes were identified after screening and eliminating duplicates (the specific 877 drug target genes were detailed in Supplementary Material (1)). Furthermore, using the OMIM and GeneCards databases, 546 and 789 GENES associated with SCDA were obtained, respectively. A total of 1,314 SCDA-related gene sets were obtained after removing duplicates and consolidating search results (Figure 1).

### 3.2. Composition-Target Network Diagram

**Figure 2:**
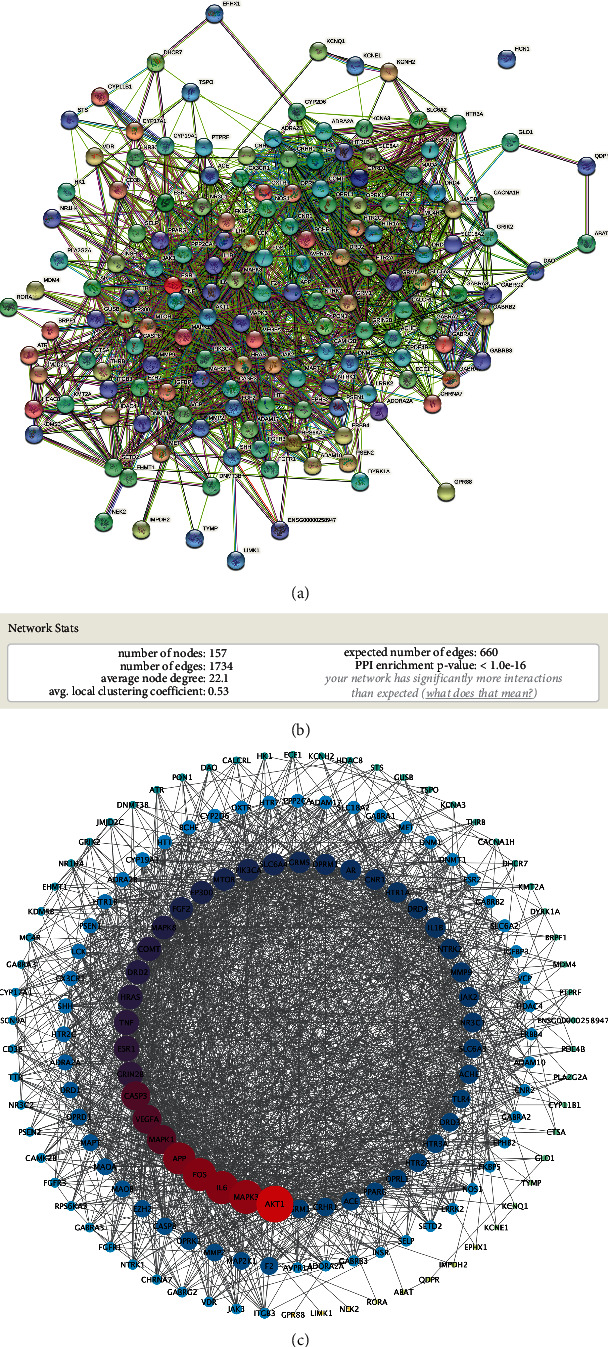
Protein-protein interaction (PPI) network: (a) the PPI network exported from the String database, (b) the annotation of nodes and edges in the PPIs network, and (c) PPIs nodes and edges as shown in Cytoscape 3.8.2.

To obtain functionally associated protein data, 157 cross-genes were uploaded to the protein interaction platform STRING 11.0, and then the target of JWYHT treatment on SCDA and the functionally related PPI networks were constructed. Cytoscape 3.8.2 displayed 157 nodes and 1,734 edges (Figure 2). The average degree value in the target was 22.1. According to the findings, AKT1 (AKT serine/threonine kinase 1) was the most targeted gene of JWYHT. As documented in National Center for Biotechnology Information (NCBI), these AKT proteins regulate a wide range of cellular functions in both normal and malignant cells, including cell proliferation, survival, metabolism, and angiogenesis.

### 3.3. PPI Network and Its Main Subnetworks

**Figure 3:**
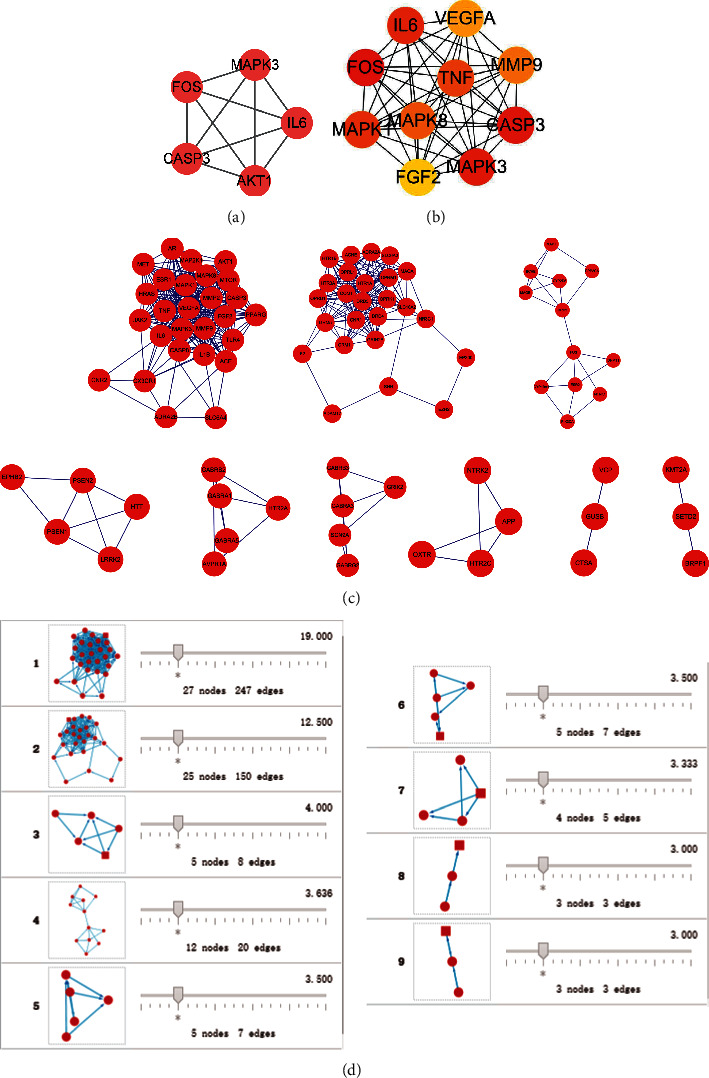
The main subnetwork: (a) the five major hub genes identified using CytoNCA plug-in, (b) the 10 most important hub genes identified by the cytoHubba plug-in, and (c–d) the nine important subnodes identified using the MCODE plug-in.

The PPIs network analysis revealed that the interaction of these target genes was complex. We imported the PPIs network into Cytoscape 3.8.2 and examined it further with the CytoNCA and cytoHubba plug-ins, yielding two major subnetworks of 5 or 10 genes, respectively (Figure 3). MCODE plug-in analysis yielded a total of 9 gene sets. The core genes of each gene set included AKT1, HTR1B, LRRK2, FOS, HTR2A, GABRG2, APP, VCP, and KMT2A. After extensive comparison, we identified 9 genes, including FOS, CASP3, MAPK3, IL6, MAPK1, TNF, MAPK8, AKT1, and 5HT1B as important genes.

### 3.4. Screening Results of Main Active Ingredients

**Figure 4:**
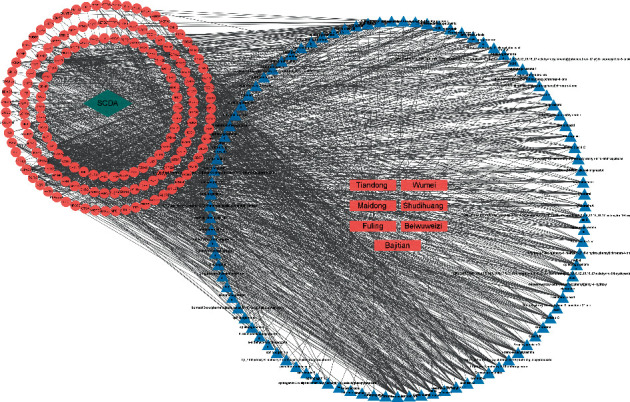
Drug-component-disease-target network. The rectangle represents the drug composition in JWYHT; the triangle represents the major active ingredients of drugs; and the circle represents the cross-target genes of SCDA and JWTHT.

The Cytoscape3.8.2 software established a medicine-component-disease-target network model based on shared targets according to the sorted network relationship (please refer to Supplementary Material (2) for details), for a better visual understanding of their interrelationships (Figure 4). Topological analysis revealed 99 active components (for details, please refer to Supplementary Material (3)), and the corresponding degree was assigned. As the degree increased, so did the correlation in the relational network. Using the twofold mean of degree as the screening condition, nine major active ingredients with a degree value greater than 25.2 were obtained, including orchinol, jasmololone, cerevisterol, 7-methoxy-2-methyl isoflavone, methyl arachidonate, gomisin G, Schizandrer B, deoxyharringtonine, and gomisin A. The above-mentioned active ingredients were used as molecular ligands in a molecular docking analysis with major gene target proteins.

### 3.5. Gene Ontology (GO) Functional Enrichment Analysis

**Figure 5:**
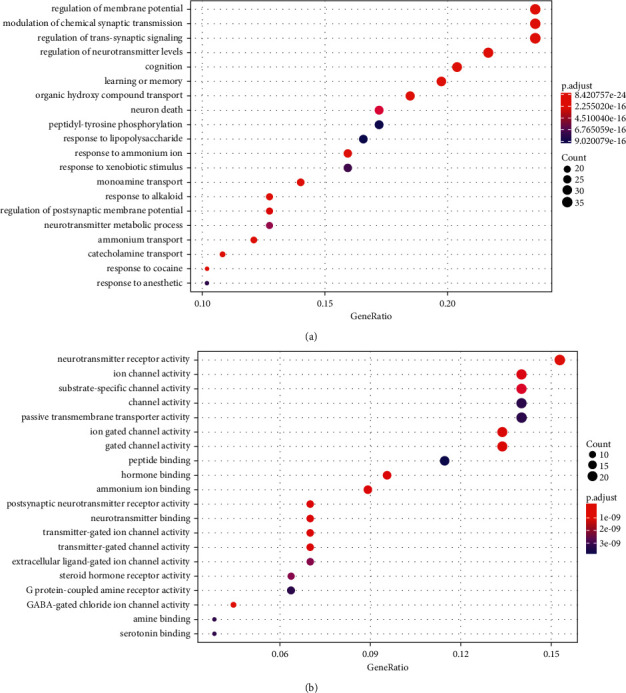
Cross target gene GO enrichment analysis bubble map: (a) the biological process (BP) and (b) the molecular function (MF). Gene ratio refers to the ratio of enriched genes to all target genes, and the count refers to the number of enriched genes.

GO enrichment analysis revealed 157 gene-related biological processes (BP) and molecular functions (MF); the significance levels were set to *P* < 0.05and *Q* < 0.05, respectively. In total, we found 2,008 important biological processes involving 178 molecular functions. Of note, according to *P*-value, 9 of the top 20 biological processes were involved in synaptic regulation. GO nomenclatures such as neurotransmitter receptor activity, ion channel activity, substrate-specific channel activity, channel activity, and passive transmembrane transporter activity were primarily involved in molecular function (Figure 5). These target genes were found to be involved in synaptic signaling and ion channel function.

### 3.6. Kyoto Encyclopedia of Genes and Genomes (KEGG) Pathway Analysis and KEGG-Target Network

**Figure 6:**
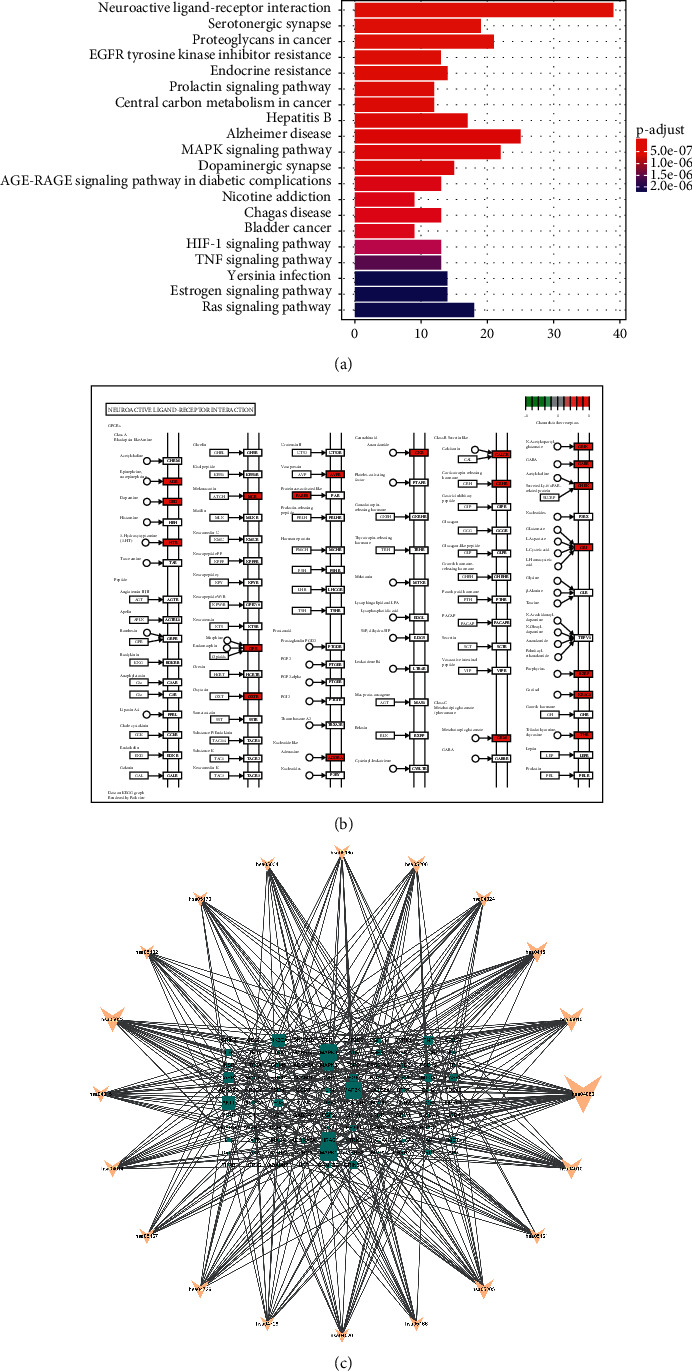
KEGG enrichment analysis and pathway mapping: (a) KEGG enrichment analysis of the target gene. Gene ratio refers to the ratio of enriched genes to all target genes. Count refers to the number of enriched genes. (b) The critical enrichment pathway involved in the neuroactive ligand-receptor interaction. (c) The schematic diagram of the KEGG gene network. The bigger the rectangle, the more complex the pathway in which the gene is involved.

KEGG enrichment analysis identified 157 gene enrichment pathways using filter waves of *P* < 0.05 and *Q* < 0.05. Finally, 150 metabolic pathways were enriched, and the results demonstrated that these genes were primarily involved in neuroactive ligand-receptor interaction, pathways of neurodegeneration – multiple diseases, Alzheimer's disease, signal transduction-related pathways, proteoglycans in cancer, serotonergic synapse, dopaminergic synapse, alcoholism, viral infection, and other pathways. Additionally, we linked KEGG-enriched pathways and cross-target genes and imported them into Cytoscape 3.8.2 to create visualizations of the major targets and pathways of JWYHT in SCDA treatment (Figure 6).

### 3.7. Molecular Docking Results

**Figure 7:**
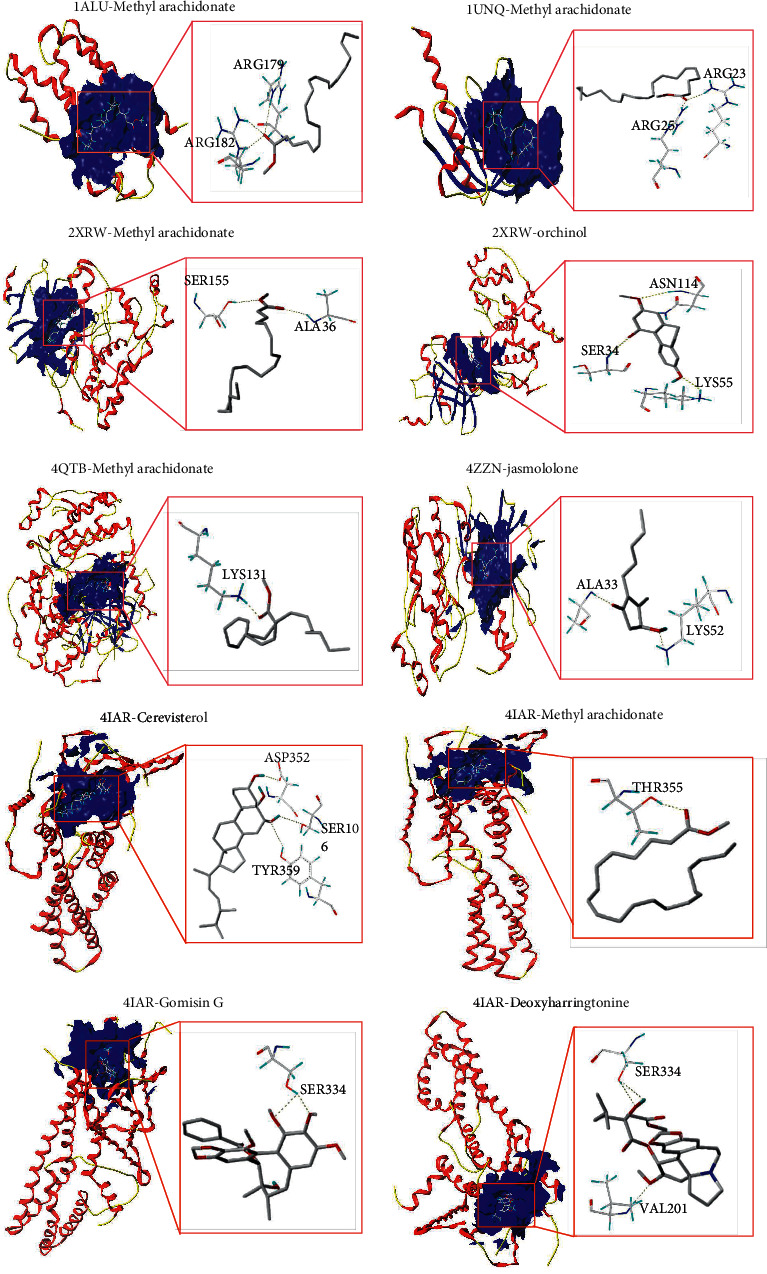
Molecular docking patterns of major ingredients and key gene targets of JWYHT. Molecular docking between a target protein with a total score of >6 and its ligand. The three-dimensional structure of the receptor surface and ligand is shown on the left, while the ligand and receptor are shown on the right.

We performed molecular docking analysis on nine major active ingredients and nine important gene target proteins to assess the reliability of core proteins targeted by JWYHT. The compound CIDs of the nine main active ingredients and the PDB IDs of the nine gene target proteins were downloaded (Tables 1 and 2).  Table 3 summarizes the docking findings. Except for 2XRW-gomisin A, 2XRW-Schizandrer B, 2XRW-gomisin G, and 4QTB-gomisin G, the total score for the remaining core ingredients and the core target were greater than 0, indicating that they had binding activity. Methyl arachidonate had docking scores higher than 8.5 with MAPK8, MAPK3, and MAPK1, showing the strongest binding activity. Notably, MAPK1 also showed good binding ability with deoxyharringtonine and jasmololone with docking scores higher than 6.0. Secondly, 5HT1B also had docking scores higher than 6.0 with methyl arachidonate, cerevisterol, deoxyharringtonine, and gomisin G, indicating strong binding activity. Also, IL6, AKT1, and methyl arachidonate can bind well.  Figure 7 depicts the molecule and protein binding sites of 2XRW-methylarachidonate, 2XRW-orchinol, 1ALU-methyl arachidonate, 1UNQ-methyl arachidonate, 4ZZN-jasmololone, 4QTB-methylarachidonate, 4IAR-cerevisterol, 4IAR-methyl arachidonate, 4IAR-gomisin G, and 4IAR-deoxyharringtonine. All of the above small molecules are stably bound to the active site of corresponding proteins through hydrogen bonding interactions with amino acids.

### 3.8. Molecular Dynamics (MD) Simulation Results

**Figure 8:**
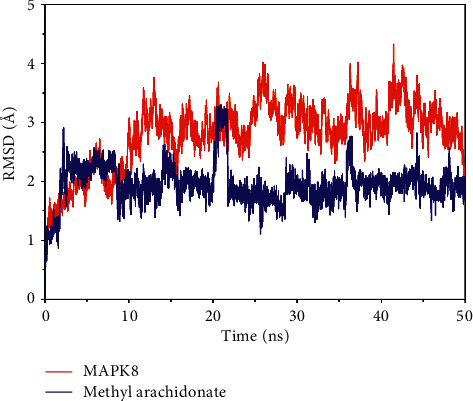
The root-mean-square deviation (RMSD) of the complex change over time during the molecular dynamics simulation.

**Figure 9:**
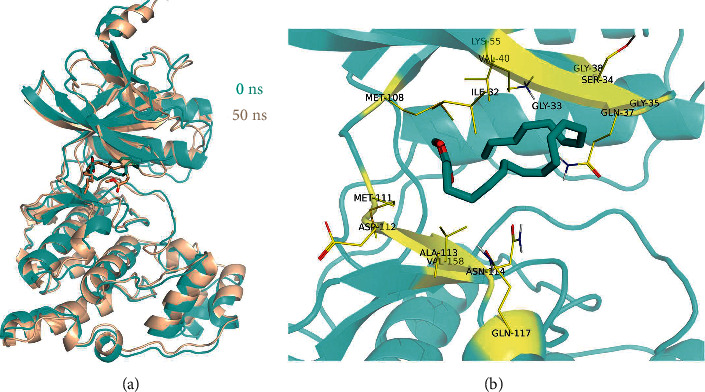
(a) The change of pose before and after MD. Wheat was the first frame of MD, and cyan was the last frame of MD. (b) The binding mode of methyl arachidonate and MAPK8. This constellation was taken from the last frame of the MD. The binding sites were mainly hydrophobic amino acids (MET-111, ALA-113, VAL-158, ILE-32, and MET-108), which were related to the van der Waals effect.

The root-mean-square deviation of a molecular dynamics simulation can reflect the movement process of the complex. Vigorous fluctuation indicates violent movement; otherwise, the movement is stable. As shown in Figure 8, the protein fluctuated smoothly, indicating that no conformational changes had occurred, and methyl arachidonate behaved smoothly in the late simulation stage. These observations indicate that methyl arachidonate had reached a convergent state and was stably bound in the MAPK8 active pocket. Furthermore, the protein conformation before and after MD remained essentially unchanged (Figure 9). However, there was a slight change in the conformation of the small molecule, which was consistent with the conclusion of the RMSD diagram. The small molecule changed in the ATP pocket early in the simulation, and it existed stably later in the simulation. The binding energy of methyl arachidonate and MAPK8 during the binding process was –30.60 ± 3.91 kcal/mol (Table 4). The binding energy was negative, indicating that it can bind to the target and thus has the potential for the biological activity of the target. Additionally, the energy decomposition shows that the van der Waals effect was as high as −41.51 ± 0.71 kcal/mol, indicating that the van der Waals effect was mainly responsible for the high binding energy of methyl arachidonate and MAPK8.

## 4. Discussion

Orchinol, jasmololone, cerevisterol, 7-methoxy-2-ethylisoflavone, methyl arachidonate, gomisin G, Schizandrer B, deoxyharringtonine, and gomisin A were identified as major active components of JWYHT by topological analysis. Methyl arachidonate, which is produced by methylating arachidonic acid (AA), demonstrated ASD-related gene binding activities. AA is an unsaturated fatty acid that aids in the improvement of cognition and the esterification of cholesterol. Monoacylglycerol lipase (MAGL) can hydrolyze 2-arachidonic acid glycerol (2-AG) into AA and glycerol, thereby regulating the endocannabinoid (ECB) system signal transduction *in vivo* and participating in the regulation of physiological processes, including pain, taste, learning, memory, and reward behavior. Inhibiting MAGL may increase the level of 2-AG in the brain while decreasing the level of AA, which improves ECB signal transduction and has an anti-neuroinflammatory effect [25]. Experimental evidence suggests that the prodrug methyl arachidonic acid may decrease endoplasmic reticulum stress and have anti-inflammatory properties [26]. Orchinol is an anti-cancer, anti-fungal, spasmolytic, anti-allergy, anti-inflammation, and anti-platelet aggregation component of the Orchidaceae family [27]. ASD risk genes may influence the gene network by regulating the RAS/ERK, PI3K/AKT, and WNT/*β*-catenin signal pathways. The degree of network dysfunction is positively correlated to ASD severity [28], and orchinol has been shown to potentially inhibit the Wnt3a/*β*-catenin signal pathway [29]. Cerevisterol (CRVS) has anti-inflammatory activity, which may decrease the inflammatory response by reducing the proinflammatory molecule IL-6 and inhibiting the MAPK signal pathway, particularly on macrophages activated by lipopolysaccharide LPS [30]. LPS is a bacterial metabolite that can easily cross the intestinal barrier and influence the inflammatory response of the brain [31], suggesting a potential mechanism of CRVS treatment of ASD by regulating intestinal flora. *S. chinensis* lignans, gomisin G, and gomisin A have neuroprotective, cognitive enhancing, and anti-inflammatory properties [32, 33]. They protect primary cortical neurons from microglia-mediated neurotoxicity by activating protein kinase A (PKA) and Nrf-2 signals while inhibiting the MAPK/STAT/NF-*κ*B pathway [34]. Its anti-inflammatory potential is achieved via anti-cyclooxygenase 1 and cyclooxygenase 2 enzyme activity [33]. *S. chinensis* refers to the dried and ripe fruit of *S. chinensis*. The primary pharmacologically active components are schisandrin A, Schizandrer B, and other biphenyclooctadiene lignans, which may improve cognitive function and anti-oxidation. Schizandrer B has been shown to increase the expression of GABAAR*α*1 and GABAAR*γ*2, thereby increasing GABA levels in peripheral brain tissue and blood [35]. Recent research has demonstrated that GABA in the temporal lobe cortex may consistently regulate the excitation and inhibition ratio of synapses and that damage to this system is linked to the core emotional, communication, and social impairment of ASD [36]. Cephalotaxus is a group of alkaloids derived from Cephalotaxus, including deoxyharringtonine, known to potentially regulate the hypothalamus-pituitary-adrenal cortex axis, inhibit TNF- *α*, and improve immune function [37]. Valproic acid-induced autistic rats were found to exhibit genotoxicity of tumor necrosis factor (TNF) *α* expression [38], implying that deoxyharringtonine may alleviate ASD by inhibiting TNF- *α*.

Following PPI network topology and cluster analysis, nine important gene target proteins, including FOS, CASP3, MAPK3, IL-6, MAPK1, TNF, MAPK8, AKT1, and 5HT1B, were identified, and AKT1 was the most targeted gene of JWYHT. AKT1 is a protein kinase B that regulates multiple cell functions, including cell growth, apoptosis, nutritional metabolism, and transcription factor activity [39]. Studies have shown that AKT1 is associated with cognitive decline and plays a role in emotional memory learning in the amygdala, which is the main area of emotional perception, whereas amygdala dysfunction causes ASD core social problems [40]. Research evidence suggests that a phosphatidylinositol 3-kinase PI3K/AKT pathway imbalance may result in ASD [41]. The AKT-mTOR signal pathway is thought to be overactive in the pathogenesis of Cntnap2-deficient ASD, and inhibiting the AKT-mTOR signal may reversibly rescue social defects in Cntnap2-/- mice [42]. MTORC1 is involved in the production of brain-derived neurotrophic factors (BDNF). mTORC1 activation is required for steady-state retrograde changes in presynaptic function [43]. In the central nervous system, BDNF can effectively regulate synaptic transmission and plasticity. BDNF-mediated ERK1/2 signal pathway regulation may increase the density of dendritic spines in hippocampal vertebral neurons and alter hippocampal-dependent learning and memory function [44]. Some experimental studies have demonstrated that JWYHT can preserve the morphology of hippocampal neurons [45], and the hippocampus is a major component of the Papez loop, which is the neuroanatomical basis of different emotions, including emotional form, instinctive correlation, and so on. Furthermore, the MET signal is involved in neocortical and cerebellar growth and maturation, immune function, and gastrointestinal repair, and it is found in a specific population of projection neurons in the neocortex and limbic system structures (including the amygdala, hippocampus, and septum) [46]. Also, its C allele genetic deletion is associated with ASD [47]. The cumulative effect of the AKT gene on MET causes the AKT/MET cascade to have a significant impact on human facial expression perception. Mounting evidence shows that facial expression detection is critical for human social interaction [48–50]. FOS can regulate neuronal metabolic activity. The activation balance of complementary basal ganglia pathways is related to FOS expression in the dorsal striatum. Reduced activation causes repetitive ASD behavior, which includes decreased glutamatergic neurotransmitter transmission, decreased metabolic activity of neurons in the subthalamic nucleus, and decreased dendritic spine density [51, 52]. CASP3 (caspase-3) is a protease that can cleave DNA and promote cell apoptosis. CASP3-deficient mice have been revealed to exhibit decreased dopaminergic internalization tissue function, exhibiting fundamental ASD symptoms such as impaired social communication, interest restriction, and repetitive behavior [53]. Elsewhere, valproic acid-induced autistic rats exhibited genotoxicity of TNF-*α* and caspase-3 expression [38]. MAPK3, MAPK1, and MAPK8 belong to the mitogen-activated protein kinase (MAPK) family, which encodes the ERK1/2 MAP kinase. MEK activates ERK1/2, resulting in an ERK signal cascade that regulates progenitor cell proliferation and differentiation, glial cell genesis, maturation, and apoptosis. ASD and other developmental disorders have been linked to ERK pathway dysfunction. Genetic evidence shows a strong link between the activation of this pathway and cognitive performance [54]. Animal studies have also revealed that the frontal cortex of ASD mice abnormally regulates Ras/Raf/ERK1/2 signal transduction [55]. ERK2 knockout causes abnormal ASD-like social behavior and long-term memory impairment [56]. CD4^+^ T cells regulate oxidative stress by releasing inflammatory factors such as IL-6. Transduction of the IL-6 receptor promotes the release of IL-17A and polarizes Th0 cells to Th17 cells. Also, the upregulation of Th17 cell development is related to ASD pathogenesis [57]. TNF is a well-studied cytokine that regulates immune regulation, brain development, cell survival/apoptosis, neurogenesis, and cell differentiation. Studies have shown that TNF can regulate critical processes related to the pathophysiology of ASD, and its elevation contributes to changes in neural connectivity, information processing, and socialization [58].

The biological regulatory mechanism of GO enrichment includes neurotransmitter receptor activity, ion channel activity, substrate-specific channel activity, channel activity, passive transmembrane transporter activity, and so on. The molecular process of synaptic transmission, which includes the coupling of electrical and chemical signals, is required for the integration of learning and memory functions. Membrane potential is essential in nerve cell communication. The movement of important ions across the membrane is required for the generation of membrane potential. CaV2 and NaV1.2 channels are related to the action potential of cortical neurons, which may tune dendritic excitability and synaptic plasticity. Both mutations and polymorphisms can be used as abnormal ASD targets, whereas CaV2 channel abnormalities can cause ASD learning or memory loss [59, 60]. Increased membrane potential causes abnormal GABA signal transduction, which may result in social interaction disorders in children with ASD [61]. Presynaptic neurotoxins influence synaptic properties by interacting with a range of postsynaptic ligands, including glial proteins, cerebellar/GluD complexes, and lipophilic proteins, thereby changing the input/output relationship of their resident neural circuits. Many mental disorders, including ASD, have been linked to gene mutations that code for neurotoxins and their ligands [62]. Neurobeachin belongs to the AKAP protein family. It binds to neurotoxins and regulates neurotransmitters released as a synaptic cell adhesion molecule, resulting in synaptic activity [63, 64]. Furthermore, neurobeachin knockdown may increase the amount of brain-derived neurotrophic factor (BDNF). The Trk-B receptor-mediated BDNF/Trk-B signal may directly activate Na^+^ and Ca2^+^ channels as well as the ERK signal pathway, thereby improving GABA neurotransmitter transmission [65]. The GABA system in the temporal cortex may regulate the excitation and inhibition ratio of synapses, and disruption to this system may be linked to core emotion, communication, and social dysfunction of ASD [36]. By modulating PI3K/AKT activity, AKAP may recruit cAMP-dependent PKA to regulate AKT phosphorylation and generate synaptic dopamine signal transduction. Additionally, neurotoxins are important regulators of presynaptic calcium channels and GABA receptor signal pathways. The aforementioned could be used as the molecular mechanism of neuronal transmission in ASD patients [66–69], and the GABA energy system described above is the main signal system in the regulation of the gut-brain axis. Some researchers have demonstrated that lactic acid bacteria and bifidobacteria in the intestine can activate GABA, transmit it to neurons, and influence the central nervous system [70–72].

The KEGG enrichment pathway is primarily made up of neuroactive ligand-receptor interactions, serotonergic synapse, and other elements. In the early stages, the fetus/cortex is the most significant developmental stage/brain area, and the interaction between neuroactive ligand-receptor and chr16q22 is the main pathway of ASD [73]. Finnish scholars discovered a significant number of rare large (>1 Mb) CNV and rare exon CNV in ASD patients in the control experiment, the majority of which belong to neuroactive ligand-receptor interaction pathways and calcium signaling pathways [74]. Similarly, according to animal studies, the neuroactive ligand-receptor interaction pathway and genes related to synaptic plasticities such as long-term potentiation, gap junction, and axon guidance are the most significant disruption pathways in ASD [75]. Some researchers confirmed the downregulation of the genes Taar7h and Taar7b in the neuroactive ligand-receptor interaction pathway using gene expression characteristics analysis and weighted gene coexpression network analysis, demonstrating that Taars and related gene regulatory networks may play an important role in ASD [76]. Recent research indicates that the novel biomarker miRNA may influence the process and etiology of ASD by regulating different genes in a variety of ways, including neuroactive ligand-receptor interactions, serotonin synapses, and so on [77]. Serotonin, also known as serotonin (5-HT), is a critical neurotransmitter produced in intestinal chromaffin cells by TPH1 and is found in the gastrointestinal tract. Evidence shows that microflora is critical in the regulation of host 5-HT [78]. Previously, a study found that BNDF levels in the hippocampus of sterile mice decreased while they increased in infected mice. Aseptic mice showed regional changes in the expression of GABA receptor An and B subunits, NMDA receptor subunits, 5-HT, tryptophan, and tryptophan metabolites [79]. 5-HT is involved in the formation of dendrites, synaptic spines, and synapses in the cerebral cortex and hippocampus in the nervous system [80], as well as the formation of cerebellar cortical networks, whereas most ASD is associated with cerebellar malformations [81]. In addition, 5-HT either directly or indirectly regulates neuronal presynaptic balance by modulating GABA energy transmission. Rat experiments have shown that 5-HT1A and 5-HT1b receptor agonists improve ASD social behavior defects by increasing serotonin activity and regulating presynaptic excitatory neurotransmission [82–84].

Therefore, JWYHT can regulate ASD social communication disorders through multiple targets and pathways, primarily by regulating signal pathways such as neuroactive ligand-receptor interaction, MAPK, AKT, and synaptic transmission of 5-HT and GABA, as well as the transmission process of gut-brain axis-related neurons and inflammatory response. These regulatory changes alter brain neuronal development, synaptic transmission, intestinal, and cerebral inflammatory response to improve ASD social behavior and related memory, emotion, and language ability either directly or indirectly. The results of molecular docking revealed that methyl arachidonate had a high affinity for MAPK8, MAPK3, and MAPK1. The MAPK pathway is involved in the regulation of autistic brain neurodevelopmental processes such as the formation of hippocampal neuron dendritic spines and synapses [85, 86], social behavior, and gastrointestinal disorders [87]. Together with the mTOR pathway, it can also regulate the severity of autism [88]. Based on the findings of the preceding studies, the MAPK pathway has been identified as a potential therapeutic target for neurodevelopmental disorders such as autism. Molecular dynamics simulation studies have demonstrated the super binding activity and stability of methyl arachidonate and MAPK8, predicting a potential key role for methyl arachidonate targeting MAPK8 in SCDA therapy. In order to provide a more solid scientific foundation, the above findings must be confirmed in the future through some *in vivo* studies.

## 5. Conclusions

The main targets of JWYHT on social communication disorders in autism, including MAPK, AKT1, 5HT1B, and IL6, were investigated using network pharmacology and molecular docking technology in this study. The most important MAPK pathway in autism is involved in neurodevelopmental disorders. Combined with molecular dynamics simulation research, MAPK8 may be one of the key targets of JWYHT in SCDA treatment. The findings of this study demonstrated the potential efficacy and molecular mechanism of traditional Chinese medicine in the treatment of autism-related social communication disorders.

## Figures and Tables

**Table 1:** The compound CID of each active ingredient obtained from the PubChem database.

Ingredients	Compound CID
Methyl arachidonate	6421258
Deoxyharringtonine	285342
7-Methoxy-2-methyl isoflavone	911486
Gomisin G	14992067
Schizandrer B	5318785
Jasmololone	5374699
Gomisin A	3001662
Cerevisterol	10181133
Orchinol	181686

**Table 2:** The ID of each gene target protein obtained from the PDB database.

Gene targets	PDB ID
FOS	1A02
IL6	1ALU
AKT1	1UNQ
CASP3	2KDO
MAPK8	2XRW
MAPK3	4QTB
MAPK1	4ZZN
TNF	5UUI
5HT1B	4IAR

**Table 3:** The total score table of the molecular docking of nine active ingredients and nine gene target proteins (binding free energy value ∆*G*0 = −2.303 RT × Total Score, where *R* denotes the ideal gas constant of the molecule and *T* denotes the thermodynamic temperature of the ideal gas).

Ingredients/PDB IDs	Total score								
	1A02	1ALU	1UNQ	2KDO	2XRW	4QTB	4ZZN	5UUI	4IAR
Methyl arachidonate	3.3727	6.7179	6.0084	5.2590	9.0847	8.9395	8.4548	5.9669	7.2170
Deoxyharringtonine	3.2290	4.7971	5.4209	3.7202	5.1176	5.2812	6.1705	2.9570	6.1325
7-Methoxy-2-methyl isoflavone	2.8356	2.0870	2.9272	2.3969	4.1191	4.1017	3.1536	1.8941	3.6842
Gomisin G	2.8087	3.4407	3.6593	3.5559	−12.1776	−2.4407	2.9937	2.8342	6.1492
Schizandrer B	2.4511	2.7017	4.3371	2.8277	−3.8579	2.6506	3.6379	2.6220	5.3347
Jasmololone	2.3559	3.5699	3.6814	3.4716	4.9767	3.9994	6.0271	2.1836	4.5538
Gomisin A	1.9745	2.7240	2.0270	2.0178	−0.1142	1.0332	4.5481	2.5717	4.5585
Cerevisterol	1.6748	5.6414	4.6216	3.9348	3.4086	3.6640	4.3381	2.9828	7.6256
Orchinol	1.6172	2.0864	4.0505	2.8374	5.4097	5.2151	4.8878	1.9665	4.8267

**Table 4:** Binding free energies and energy components predicted by MM/GBSA (kcal/mol).

System name	MAPK8-methyl arachidonate
Δ*E*_vdw_	−41.51 ± 3.84
Δ*E*_elec_	−2.92 ± 0.87
ΔG_GB_	19.53 ± 0.71
ΔG_SA_	−5.70 ± 0.60
ΔG_bind_	−30.60 ± 3.91

ΔE_vdW_: van der Waals energy, ΔE_elec_: electrostatic energy, ΔG_GB_: electrostatic contribution to solvation, ΔG_SA_: nonpolar contribution to solvation, and ΔG_bind_: binding free energy.

## Data Availability

The data used and/or analyzed during the current study are available from the corresponding author upon request.

## Supplementary Materials

Supplementary Materials (1) The specific 877 drug target genes. (2) The Cytoscape3.8.2 software established a medicine-component-disease-target network model based on shared targets according to the sorted network relationship. (3) Topological analysis revealed 99 active components.
